# A Randomized Controlled Trial on the Efficacy of a Psychosocial Behavioral Intervention to Improve the Lifestyle of Patients With Severe Mental Disorders: Study Protocol

**DOI:** 10.3389/fpsyt.2018.00235

**Published:** 2018-06-07

**Authors:** Gaia Sampogna, Andrea Fiorillo, Mario Luciano, Valeria Del Vecchio, Luca Steardo, Benedetta Pocai, Marina Barone, Mario Amore, Francesca Pacitti, Liliana Dell'Osso, Giorgio Di Lorenzo, Mario Maj, LIFESTYLE Working Group, G. Borriello

**Affiliations:** ^1^Department of Psychiatry, University of Campania “Luigi Vanvitelli”, Naples, Italy; ^2^Department of Basic Medical Science, Neuroscience and Sense Organs, University of Bari “Aldo Moro”, Bari, Italy; ^3^Department of Neuroscience, Rehabilitation, Ophthalmology, Genetics, Maternal and Child Health, Section of Psychiatry, University of Genoa, Genoa, Italy; ^4^Section of Psychiatry, Department of Biotechnological and Applied Clinical Sciences, University of L'Aquila, L'Aquila, Italy; ^5^Department of Clinical and Experimental Medicine, University of Pisa, Pisa, Italy; ^6^Department of System Medicine, University of Rome Tor Vergata, Rome, Italy

**Keywords:** mortality gap, mental disorders, lifestyle, diet, physical activity

## Abstract

Patients with severe mental disorders die on average 20 years prior to the general population. This mortality gap is mainly due to the higher prevalence of physical diseases and the adoption of unhealthy lifestyle behaviors.The LIFESTYLE trial aims to evaluate the efficacy of a new psychosocial group intervention (including psychoeducational, motivational, and problem-solving techniques) focused on healthy lifestyle behavior compared to a brief educational group intervention in a community sample of patients with severe mental disorders. The trial is a national-funded, multicentric, randomized controlled trial with blinded outcome assessments, which is carried out in six outpatient units of the Universities of Campania “Luigi Vanvitelli” in Naples, Bari, Genova, L'Aquila, Pisa, and Rome—Tor Vergata. All patients are assessed at the following time points: baseline (T0); 2 months post-randomization (T1); 4 months post-randomization (T2); 6 months post-randomization (T3); 12 months post-randomization (T4); and 24 months post-randomization (T5). T1 and T2 assessments include only anthropometric tests. The BMI, a reliable and feasible anthropometric parameter, has been selected as primary outcome. In particular, the mean value of BMI at 6 months from baseline (T3) will be evaluated through a Generalized Estimated Equation model. The work hypothesis is that the LIFESTYLE psychosocial group intervention will be more effective than the brief educational group intervention in reducing the BMI. We expect a mean difference between the two groups of at least one point (and standard deviation of two points) at BMI. Secondary outcomes are: the improvement in dietary patterns, in smoking habits, in sleeping habits, physical activity, personal and social functioning, severity of physical comorbidities, and adherence to medications. The expected sample size consists of 420 patients (70 patients for each of the six participating centers), and they are allocated with a 1:1 ratio randomization, stratified according to center, age, gender, and educational level. Heavy smoking, sedentary behavior, and unhealthy diet pattern are very frequent and are associated with a reduced life expectancy and higher levels of physical comorbidities in people with severe mental disorders. New interventions are needed and we hope that the LIFESTYLE protocol will help to fill this gap.

Trial registration number: 2015C7374S.

## Introduction

In the last decade, the need to improve physical health in patients with severe mental disorders, namely schizophrenia, major depression, and bipolar disorders (1), has been repeatedly highlighted by several international scientific bodies and reported in several guidelines (2–6). Nevertheless, physical health of people with several mental disorders is often neglected by the patients themselves, as well as by their caregivers and mental health professionals (1).

Patients with severe mental disorders die on average 20 years prior to the general population (7). In patients with severe mental disorders, cardiovascular diseases, and other physical diseases contribute significantly to the reduced life expectancy, even if more than other “unnatural” causes, such as accidents or suicide (8–10). Among the relevant and modifiable factors for explaining the high cardiovascular morbidity and mortality of patients with severe mental disorders two factors have been repeatedly recognized: the poor access to prevention and screening programmes and the adoption of unhealthy lifestyle behaviors (11). It is unacceptable that life expectancy, which is substantially grown in developed countries in the general population, remains so short in people with schizophrenia and other severe mental disorders (12, 13).

Recently, it has been reported that receiving a diagnosis of schizophrenia is associated with at least 13–15 years of potential life lost; therefore, there is the need to develop and implement individual-based and/or community-level interventions in order to reduce this excess mortality in people with severe mental disorders (1, 14).

Interventions could improve global health and well-being in people with severe mental illness by modifying their unhealthy lifestyle behaviors. According to a recent review, people with mental disorders are more sedentary than the general population (matched for gender and age) and are significantly less physically active. Moreover, at least 50% of people with severe mental illness do not meet the general recommendation of performing at least 150 min of moderate physical activity per week (1). Moreover, patients with severe mental disorders have unhealthy diet pattern, with a low intake in fruit and fiber and a high intake of junk food (15, 16). Compared with the general population, people with mental disorders have a higher probability of being heavy smokers (i.e., more than two packs/day) (17–19).

Increasing the levels of physical activity, improving dietary patterns, and reducing smoking habits of people with severe mental disorder may represent a global health challenge and a public health priority.

On these premises, several psychosocial interventions—including behavioral, educational, and psychological components—have been developed worldwide for addressing these needs (20–25). In particular, some interventions have been specifically focused on improving dietary patterns [e.g., (24)] or smoking habits [e.g., (26)] or on physical inactivity, unhealthy dietary habits, and smoking [e.g., (22, 23)]. These lifestyle psychosocial interventions—that are different in format, setting, duration, and involved professionals—have been found to be effective in improving patients' physical health.

However, several aspects have been identified as critical for the provision of lifestyle interventions in the clinical routine care. The optimal duration of the intervention; the involvement of a multidisciplinary team: the evaluation of cost/effectiveness ratio: the feasibility of the intervention in the real word practice are some of the most crucial ones. Recently, Speyer et al. (22, 23) found that the CHANGE trial was not superior to treatment as usual in reducing cardiovascular risk in patients with schizophrenia spectrum disorders and abdominal obesity, but more data are needed for patients with other severe mental disorders.

Moreover, there is the need to understand if these interventions actually reduce the mortality gap, and they should be evaluated in routine care, in large samples and in the long term (25). In order to assess the efficacy and the feasibility of these lifestyle interventions, an easy, reliable, and affordable anthropometric parameter, as the Body Mass Index (BMI) should be selected (15, 16, 24). Under these premises, a multicentric collaborative study, coordinated by the Department of Psychiatry of the University of Campania “Luigi Vanvitelli in Naples, Italy” and funded by the Italian Ministry of Education, Universities and Research, has been proposed for evaluating the efficacy and effectiveness of new psychosocial group interventions targeted on lifestyle in a real-world sample of patients with severe mental disorders, in terms of reduction of BMI.

## Aims

The LIFESTYLE trial aims to evaluate the efficacy of a new psychosocial group intervention focused on healthy lifestyle behaviors compared to a psychoeducational brief group intervention in a community sample of patients with severe mental disorders on several variables including BMI, comorbidity index, smoking habits, and dietary patterns.

The secondary aims of the trial are: (1) to identify predictive factors associated with poor response to the interventions; (2) to define the role of mediators and moderators for the efficacy of the interventions; (3) to describe differences according to diagnostic groups in terms of improvement of lifestyle behaviors; (4) to evaluate the long-term effects of the interventions and their cost-effectiveness; (5) to develop guidelines for improving healthy lifestyle in people with severe mental disorders in the clinical routine care.

## Methods

### Design

The LIFESTYLE trial is a national-funded, multicentric, randomized controlled trial with blinded outcome assessments. The project has been funded by a grant of the Italian Ministry of Education, Universities and Research within the framework of the “Progetti di Rilevante Interesse Nazionale (PRIN) — year 2015”. It is a multicentric study involving the outpatient units of the University of Campania “Luigi Vanvitelli” in Naples, University of Bari, University of Genova, University of L'Aquila, University of Pisa, and University of Rome—Tor Vergata. The University of Campania “Luigi Vanvitelli” in Naples is the coordinating center, which has originally conceived the study's idea and design.

This is a randomized head-to-head comparison trial with two parallel arms for evaluating the efficacy and effectiveness of psychosocial group interventions in improving lifestyle behaviors in patients with severe mental disorders. The two interventions have been developed by the research staff and are both focused on the improvement of lifestyle behaviors.

### Patients' recruitment

Patients are being recruited in six outpatient units from the universities of Naples, Bari, Genova, L'Aquila, Pisa, and Rome—Tor Vergata.

Eligible patients are identified by their clinicians and referred to the LIFESTYLE research staff by phone, email, or in-person contact. After the patients have been contacted, a meeting is arranged at the outpatient clinic with the referring clinician and the LIFESTYLE staff. Verbal and written information about the project are provided. If the patient accepts to participate to the study, an informed consent form is signed and an appointment for baseline assessment is made. Once the informed consent is collected, the patient is formally enrolled in the study's protocol. Before starting the baseline assessment, the LIFESTYLE staff member contacts the statistician working at the Coordinating Center for obtaining the randomization code for patients' group allocation. The randomization procedure is stratified according to center, age, gender, and educational level with a 1:1 ratio. The treating psychiatrist will continue to provide the usual treatment to patients.

### Patients' inclusion criteria

Patients have to fulfil the following inclusion criteria:

- Age between 18 and 65 years.
- Diagnosis of schizophrenia, schizoaffective disorder, delusional disorder, other psychotic disorders, major depressive disorder, or bipolar disorder according to the DSM-5 and confirmed by the Structured Clinical Interview for DSM-5 (SCID-5) (27).
- Ability to provide written informed consent.
- BMI ≥ 25.

### Patients' exclusion criteria

Patients are excluded from the study if they meet one of the following exclusion criteria:

- Inability to perform moderate physical activity (i.e., walking at least 150 min per week, or 75 min of vigorous activity twice a week, according to the guidelines of the Italian Ministry of Health).
- Pregnancy or breast-feeding.
- Intellectual disability or severe cognitive impairment.
- Worsening of clinical status or hospital admission in the previous 3 months.

### Blinding

Researchers and statisticians involved in patients' assessments are blinded to patient's allocation. Patients and health professionals providing the interventions are not blinded to patient allocation.

## Interventions

### Arm I: lifestyle psychosocial group intervention

The theoretical background of the new psychosocial group intervention includes techniques derived from classic psychoeducation (28), motivational intervention (29–31), and cognitive-behavioral therapy (32).

The intervention has been developed following the guidelines on the management of physical health in people with mental disorders produced by the World Health Organization (33, 34), the European Association for the Study of Diabetes (35), the European Society of Cardiology (36), and the European Psychiatric Association (37).

The adopted methodology included the following phases: (1) analysis of the scientific literature; (2) evaluation of available handbooks and manuals on other psychosocial interventions targeting lifestyle behaviors; (3) focus groups with expert researchers and clinicians, and with users and carers, in order to identify the most relevant needs to be included in the intervention; (4) development of *ad-hoc* manuals by the research group with a detailed description of each session of the intervention. Manuals have been developed in order to ensure treatment fidelity among the different centers. Leaflets and other written materials are given to patients, whenever relevant.

The intervention lasts 5 months and it is administered to groups of 5–10 patients every 7–10 days. The sessions cover the following topics: (1) healthy diet; (2) physical activity; (3) smoking habits; (4) medication adherence; (5) risky behaviors; (6) promotion of circadian rhythms. Each module includes the following components: (a) information on the risks of the specific unhealthy lifestyle and benefits in adopting healthy lifestyle; (b) teaching of practical strategies to change unhealthy behaviors; (c) identification of personal life goals for each participant, motivation to change, and problem-solving strategies. Sessions have been developed in order to stimulate discussion, work-groups and interaction among participants. At the end of each meeting, a 20-min session of moderate physical activity is scheduled (Table 1). The core feature of this intervention is the inclusion of the motivational component. During each session, group participants are supported by mental health professional in identifying a personal healthy lifestyle goal. After goal-definition, professionals help participant to define the motivations for lifestyle changing and teach problem-solving strategies for sustaining the behavioral change.

**Table 1:** Programme of LIFESTYLE psychosocial group intervention.

Starting session: Introduction of the intervention, aims, purposes, presentation of participants, and definition of personal healthy lifestyle goals
Module 1: Diet, information on health food, principles, and benefits of healthy eating
Module 2: Physical activity, how to increase routine physical activity
Module 3: Smoking habits, information on the dangers of smoking, craving, difficulties for quitting smoking and consequences of long-term abuse of nicotine.
Module 4: Medication adherence, strategies for improving adherence, medical consequences of non-adherence
Module 5: Risky behaviors, sexually transmitted disorders, substance, and alcohol abuse
Module 6: Promotion of regular circadian rhythm, problems related to irregular daily activities
At the end of each meeting, a 20-min session of moderate physical activity (i.e., walking) is implemented with participants
Information and leaflets are provided to participants during each session. Positive changes are highlighted, potential strategies for change are discussed at each module. The core feature of the intervention is the inclusion of the motivational component. During each session, group participants are supported by mental health professionals in identifying a personal healthy lifestyle goal. After goal-definition, professionals help participant to define the motivations for lifestyle changing and teach problem-solving strategies for sustaining the behavioral change

### Arm II: brief educational group intervention

The brief educational group intervention, which includes elements from the psychoeducational approach and from problem-solving techniques, consists of 5 weekly sessions and it is administered to groups of 5–10 patients. The following topics are covered: (1) healthy lifestyle; (2) early detection of clinical relapses; (3) effects of pharmacological treatment and management of side effects; (4) stress management techniques; (5) problem-solving techniques.

Manuals have been developed in order to ensure treatment fidelity among the different centers. Leaflets and other written materials are given to patients, whenever relevant. During the sessions, interaction among participants is supported using role-plays and work-groups (Table 2).

**Table 2:** Programme of LIFESTYLE educational brief group intervention.

Starting session: Introduction of the intervention, aims, purposes, and presentation of participants
Module 1: Healthy lifestyle (e.g., healthy diet, physical activity, smoking habits, promotion of circadian rhythm)
Module 2: Early detection of psychiatric relapses
Module 3: Pharmacological treatment and management of side effects
Module 4: Stress management techniques
Module 5: Problem-solving techniques
Information and leaflets are provided to participants during each session. Positive changes are highlighted, potential strategies for change are discussed at each module. The main aim is to provide patients with the principles of healthy living (i.e., eating fruit and vegetable, drinking water, doing moderate physical activities, not abusing of alcohol, quit smoking, etc.)

## Training of mental health professionals

For each participating center, three mental health professionals (at least one being a psychiatrist) participated to a 5-day training course for the provision of the two interventions, held in Naples in June 2017. Supervision meetings are organized during the study period as well as regular phone and e-mail supervisions.

## Ethical issues

This study is being conducted in accordance with globally accepted standards of good practice, in agreement with the Declaration of Helsinki and with local regulations.

The study investigators ensure that all mental health professionals involved in the study are qualified and informed about the protocol, interventions, and trial-related duties. The coordinating center has a list of all qualified mental health professionals involved in the study.

A formal ethical approval for conducting the trial has been obtained by the Coordinating Center's Ethics Committee, which approved the whole study protocol on January 2017 (approval number: prot. 64).

## Assessment time and instruments

Researchers participating to the study are blinded to patient allocation. All patients are assessed at the following time points: baseline (T0); 2 months post-randomization (T1); 4 months post-randomization (T2); 6 months post-randomization (T3); 12 months post-randomization (T4); and 24 months post-randomization (T5). T1 and T2 assessments include only anthropometric tests. All data will be collected through a paper and pencil interviewing and the expected time for filling in all assessment tools is about 60/90 min. During the completion of the assessment battery, whether patients feel distressed or exhausted, the assessment can be stopped and another appointment will be scheduled in the next 2/3 days with the researcher.

The following questionnaires and schedules will be used during the study (Table 3):

- The Food Frequency Questionnaire—short version (38) is a 18-item questionnaire on the frequency of consumption of a variety of foods corresponding to one's usual diet. In order to compile the questionnaire, the Scotti-Bassani Atlas is used for selecting quantity of food.
- The International Physical Activity Questionnaire (IPAQ)—short form (39) is a 18-item questionnaire exploring physical activity in terms of time spent in walking, in moderate-intensity and vigorous-intensity activities.
- The Fagerström Test for Nicotine Dependence (FTND) (40) is a 6-item questionnaire assessing the intensity of physical addiction to nicotine in terms of number of cigarette smoked per day, compulsion to use, and nicotine addiction.
- The Pittsburgh Sleep Quality Index (PSQI) (41) is 19-item questionnaire assessing sleep quality over a 1-month time interval.
- The Leeds Dependence Questionnaire (LDQ) (42) is a 10-item questionnaire designed to measure dependence for a variety of substances.
- The Morisky Medication Adherence Scale (MMAS) (43) is a 4-item questionnaire for evaluating adherence to pharmacological treatments.
- The Cumulative Illness Rating Scale (CIRS) (44) is a 14-item questionnaire including a comprehensive assessment of physical comorbidities.
- The Manchester Short Assessment of Quality of Life (45) is a 17-item questionnaire assessing quality of life focusing on satisfaction with life as a whole and with life domains.
- The Recovery Style Questionnaire (RSQ) (46) is a 39-item self-report questionnaire exploring six styles of adaptation to severe mental disorder and recovery: sealing over, tends toward sealing over, mixed picture in which sealing over predominates, mixed picture in which integration predominates, tends toward integration, and integration.
- The Measurement and Treatment Research to Improve Cognition in Schizophrenia (MATRICS) Consensus Cognitive Battery (MCCB)—brief version, which includes the MATRICS Consensus Trail Making Test—part A, Brief Assessment of Cognition in Schizophrenia: Symbol Coding, Category Fluency-Animal Naming (47, 48).
- Internalized Stigma of Mental Illness (ISMI), a 29-item questionnaire for evaluating the experience of stigma and internalized self-rejection (49). Each item is rated on a 4-level Likert scale, where higher scores indicate greater levels of internalized stigma.
- Questionnaire on lifestyle behaviors, a 24-items questionnaire, developed by the Italian National Institute of Health, which evaluates dietary patterns (e.g., food eaten at breakfast or lunch time), smoking habits (e.g., numbers of cigarettes smoked per day; attempts to quit smoking), and physical activity (e.g., time spent in walking per day) (50).
- The Questionnaire on sexual health, it is a 13-item *ad-hoc* questionnaire developed by the research team, which evaluates sexual behaviors and attitudes.
- The Pattern of Care Schedule (PCS)—modified version (51), a 40-item questionnaire on pharmacological and non-pharmacological treatments as well as on health care access made by the patient. It is compiled by the researcher in collaboration with the patient. If information is inadequate, or if the researcher is not sure about patients' reliability, other sources (e.g., treating physician, relatives, etc.) can be consulted. During the study period, the treating clinician will continue to provide the usual treatment to patient, and—if necessary—to change or adjust the pharmacological regimen.
- The anthropometric schedule is compiled by the researcher for collecting information on weight, height, BMI, waist circumference, blood pressure, resting heart rate, HDL, LDL, and overall level of cholesterol, blood glucose, triglycerides, and blood insulin. Moreover, the homeostatic model assessment (HOMA) index will be calculated for quantifying insulin resistance and beta-cell functioning as well as the Framingham Risk Score, for evaluating cardiovascular risk.

**Table 3:** Assessment tools and time-points of the evaluation.

**Assessment tool**	**Domains covered**	**Assessments' timepoints**					
		**T0 (baseline)**	**T1 (2 months)**	**T2 (4 months)**	**T3 (6 months)**	**T4 (12 months)**	**T5 (24 months)**
Pattern of Care Schedule (PCS)—modified version	A 40-item questionnaire on pharmacological and non-pharmacological treatments as well as on health care access made by the patient. It is compiled by the researcher in collaboration with the patient. If information is inadequate, or if the researcher is not sure about patients' reliability, other sources (e.g., treating physician, relatives, etc.) can be consulted	x			x	x	x
Socio-demographic schedule	Information were collected: age, gender, marital status, level of education, working condition, economic status, number of family members, illness duration, time in charge at the mental health centre (months), number of (voluntary and involuntary) hospitalizations, suicide attempts (numbers)	x					
Anthropometric schedule	It is compiled by the researcher for collecting information on weight, height, BMI, waist circumference, blood pressure, resting heart rate, HDL, LDL and overall level of cholesterol, blood glucose, triglycerides and blood insulin. Moreover, the homeostatic model assessment (HOMA) index will be calculated for quantifying insulin resistance and beta-cell function as well as the Framingham Risk Score, for evaluating cardiovascular risk	x	x	x	x	x	x
Structured Clinical Interview for DSM-5 (SCID-5)	It is a semi-structured interview guide for making DSM-5 diagnoses. It is administered by a trained mental health professional that is familiar with the DSM-5 classification and diagnostic criteria	x					
Measurement and Treatment Research to Improve Cognition in Schizophrenia (MATRICS) Consensus Cognitive Battery—brief version	It includes the MATRICS Consensus Trail Making Test—part A, Brief Assessment of Cognition in Schizophrenia: Symbol Coding, Category Fluency-Animal Naming (45, 46)	x			x	x	x
Brief Psychiatric Rating Scale (BPRS)	It is a semi-structured 24-item interview on psychopathological status. The items are grouped in four subscales: positive symptoms, negative symptoms, depressive-anxiety symptoms, and manic-hostility symptoms	x			x	x	x
Cumulative Illness Rating Scale (CIRS)	It is a 14-item questionnaire including a comprehensive assessment of physical comorbidities	x			x	x	x
Fagerström Test for Nicotine Dependence (FTND)	It is a 6-item questionnaire assessing the intensity of physical addiction to nicotine in terms of number of cigarette smoked per day, compulsion to use, and nicotine addiction	x			x	x	x
Food Frequency Questionnaire—short version	It is a 18-item questionnaire on the frequency consumption of a variety of foods corresponding to one's usual diet. In order to compile the questionnaire, the Scotti-Bassani Atlas is used for selecting quantity of food	x			x	x	x
Internalized Stigma of Mental Illness Inventory	It is a 29-items questionnaire for evaluating the subjective experience of stigma	x			x	x	x
International Physical Activity Questionnaire (IPAQ)—short form	It is a 18-item questionnaire exploring physical activity in terms of time spent in walking, in moderate-intensity and vigorous-intensity activities	x			x	x	x
Questionnaire on sexual health	It is an ad-hoc questionnaire developed by the research team, which evaluates sexual behaviours and attitudes	x			x	x	x
Leeds Dependence Questionnaire (LDQ)	It is a 10-item questionnaire designed to measure dependence for a variety of substances	x			x	x	x
Manchester Short Assessment of Quality of Life	It is a 17-item questionnaire assessing quality of life focusing on satisfaction with life as a whole and with life domains	x			x	x	x
Morisky Medication Adherence Scale (MMAS)	It is a 4-item questionnaire for evaluating adherence to pharmacological treatments	x			x	x	x
Personal and Social Performance Scale	It is a 100-point single-item rating scale, subdivided into 10 equal intervals. The ratings are based mainly on the assessment of patient's functioning in four main areas: (1) socially useful activities; (2) personal and social relationships; (3) self-care; and (4) disturbing and aggressive behaviours	x			x	x	x
Pittsburgh Sleep Quality Index (PSQI)	It is 19-items questionnaire assessing sleep quality over a 1-month time interval	x			x	x	x
Questionnaire in lifestyle behaviours	It consists of 24 items, which evaluates dietary patterns (e.g., food eaten at breakfast or lunch time), smoking habits (e.g., numbers of cigarettes smoked per day; attempts to quit smoking), physical activity (e.g., time spent in walking per day)	x			x	x	x
The Recovery Style Questionnaire (RSQ)	It is a 39-item self-report questionnaire exploring six styles of adaptation to severe mental illness and recovery: sealing over, tends toward sealing over, mixed picture in which sealing over predominates, mixed picture in which integration predominates, tends towards integration, and integration	x			x	x	x
ECG evaluation		x					

The following semi-structured interviews will be used during the study:

- The Brief Psychiatric Rating Scale (BPRS) (52) is a semi-structured 24-item interview on psychopathological status. The items are grouped in four subscales: positive symptoms, negative symptoms, depressive-anxiety symptoms, and manic-hostility symptoms.
- The Personal and Social Performance Scale (53) is a 100-point single-item rating scale, subdivided into 10 equal intervals. The ratings are based mainly on the assessment of patient's functioning in four main areas: (1) socially useful activities; (2) personal and social relationships; (3) self-care; and (4) disturbing and aggressive behaviors.
- The Structured Clinical Interview for DSM-5 (SCID-5) is a semi-structured interview guide for making DSM-5 diagnoses. It is administered by a trained mental health professional that is familiar with the DSM-5 classification and diagnostic criteria.

The inter-rater reliability among researchers participating in the study was tested through Cohen's Kappa coefficient during the training course on the assessment instruments (54). Case-vignettes, videos and role-play have been used in order to ensure fidelity. The Cohen's kappa values were found to be satisfactory for PSP with a value of 0.918 and for the BPRS with a value from 0.835 to 0.972. For the SCID-5, 100% agreement rate was found.

## Anticipated results

### Primary outcome

BMI has been selected as primary outcome since it is a reliable and feasible anthropometric parameter, and it has already been considered in previous studies on lifestyle interventions (15, 16, 24). The primary outcome is the change of the BMI at 6 months post-randomization (T3). We expect a mean difference between the two groups of at least one point (and standard deviation of 2 points) at BMI. The work hypothesis is that the LIFESTYLE psychosocial group intervention will be more effective than the brief educational group intervention in reducing the BMI.

### Secondary outcome

The secondary outcomes include an improvement in dietary patterns (i.e., a reduction in fat food and increase in consumption of fruits and vegetables), smoking habits (i.e., a reduction in the number of cigarettes smoked per day), sleeping habits (i.e., regular number of hours slept per night), physical activity (i.e., increasing in time spent walking per day), personal and social functioning (i.e., level of functioning and time dedicated to meeting friends), severity of physical comorbidities (i.e., reduction at the CIRS global score), and adherence to medications (i.e., reduction in changes in pharmacological doses and regimen).

### Exploratory outcomes

Exploratory outcomes include anthropometric measures, such as waist circumference measured between the iliac crest and lowest rib, blood pressure measured on the right upper arm after 10 min of rest in a sitting position, resting heart rate after 10 min of rest, HDL, LDL cholesterol, blood glucose, triglycerides, blood insulin, HOMA index, and Framingham score.

Moreover, the severity of psychiatric symptomatology (as evaluated at the BPRS) and the levels of cognitive dysfunctions (as evaluated at the brief-MATRICS) were considered exploratory outcomes.

## Baseline assessments

At baseline the following socio-demographic information were collected: age, gender, marital status, level of education, working condition, economic status, number of family members, illness duration, time in charge at the mental health center (months), number of (voluntary and involuntary) hospitalizations, suicide attempts (numbers). Moreover, ECG evaluation was mandatory in order to check the eligibility to perform moderate physical activity.

## Statistical analyses

### Sample size and power calculation

A power analysis has been implemented in order to define the sample size needed to detect a significant BMI reduction (primary study's outcome) between the two groups. In particular, it has been estimated a sample size needed to observe a mean difference between the two groups of at least one point at the BMI (kg/m^2^) (with standard deviation of 2), with power set at 90% and a significant two-tailed alpha level of 0.05, based on previous studies on the same topic [e.g., (21, 23)].

**Figure 1:**
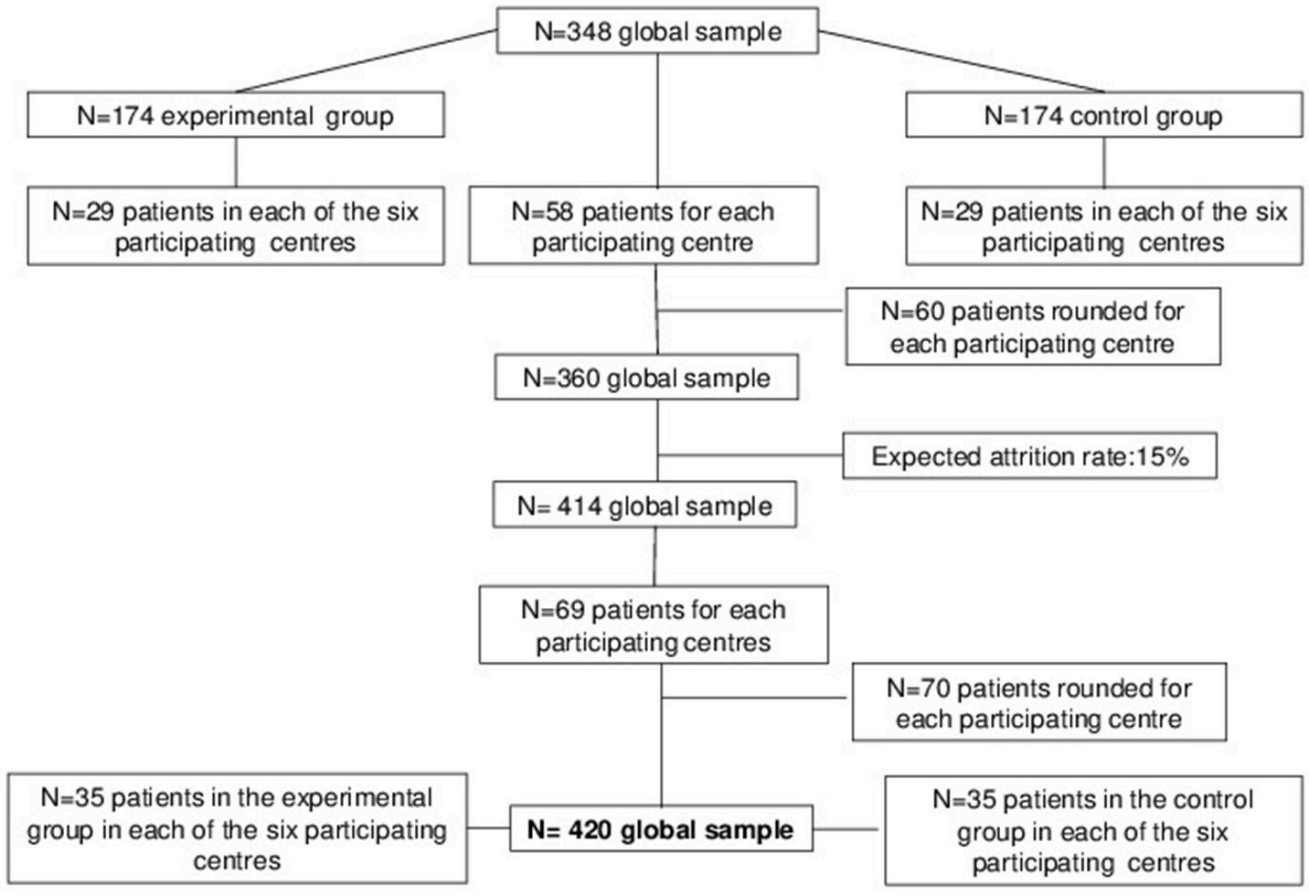
Power analysis and sample size.

Taking into account the multicenter design of the study, sample size has been adjusted for the Intercluster Correlation Coefficient (ICC), set at 0.030, and for the coefficient of variation of cluster sizes (COV), set at 0.400 (55). Therefore, the sample size consists of 348 patients (29 patients per arm in each center). Each center is expected to recruit 58 patients, rounded to 60. Moreover, previous trials evaluating the efficacy of interventions focused on physical activities and diet reported a drop-out rate of 15%; thus, the sample size has been rounded to 35 patients per arm for each center. Therefore, the expected sample size consists of 420 patients (70 patients for each participating center). This sample size has been considered feasible by all participating centers (Figure 1).

### Data analysis

Statistical analyses will be conducted according to the “Intention To Treat” principle. Missing data will be handled using the Last Observation Carried Forward (LOCF). All patients who signed informed consent form as well as those who undergo the initial assessment will be included. Descriptive statistics will be calculated for the dependant and confounding variables for both groups. The homogeneity of the two groups for these variables at baseline will be checked. In all cases, a bilateral alpha of 0.05 is considered an error and confidence intervals are calculated at 95%.

The analytic plan includes:

1. The implementation of a generalized estimated equation model (GEE). The GEE provides an estimation of the longitudinal impact of the interventions on primary and secondary outcomes, controlling for confounders, such as physical comorbidities, severity of psychiatric symptoms, level of social functioning, quality of life, self-esteem, recovery style, support from social network. A generalized estimating equation model will be employed using the following outcomes variables: the BMI, the severity index of the Cumulative Illness Rating Scale, the abdominal circumference: plasma levels of glucose, insulin, triglycerides, and cholesterol levels (total, LDL, and HDL); systolic and diastolic blood pressure, and cigarettes smoked per day.
2. The development of a Structural Equation Model (SEM) in order to identify possible mediators and moderators of the efficacy of the interventions, controlling for several confounders such as age, gender, severity of symptoms, BMI, etc. In particular, the variable “duration of the intervention” will be tested as one of the most relevant mediator or moderator for the efficacy of the interventions.
3. The implementation of multivariable logistic regression models, in order to identify predictors of positive response for changing unhealthy behaviors. In particular, as regards the primary outcome, this will be transformed in binary variable (yes vs. no) using the reduction of at least 1 point at the BMI as threshold. The impact of possible predictors (e.g., having an healthy diet; walking at least 30min per day; quitting smoking) as well as confounders (such as age, gender, level of education, diagnosis, duration of the illness, type of pharmacological treatment received) will be tested in a logistic regression model.
4. A subgroups analysis, according to the different diagnostic groups, in order to identify different profiles of efficacy of the interventions. The hypothesis is that patients suffering from affective disorders (both major depression and bipolar disorders) will report a higher improvement in the reduction of BMI and in changing their unhealthy lifestyle behaviors compared to patients with schizophrenia.

All data will be collected and analyzed by a statistician, who will be blinded during the trial. Data will be stored in hard copy by each center in a safe place. They will be placed on an online dataset, and will be accessible to each unit. For each patient, only the recognition ID will be reported in the dataset. For safety reasons and to ensure patients' privacy, the dataset will be protected by a password. Each center will have its own password, and will be responsible for data entry. A statistician from the coordinating center will manage data collection and will provide assistance to researchers in case of difficulties in using the software. It will be possible to export data in compatible formats with common calculation software (e.g., Microsoft Access and Excel) and in specific software (e.g., SPSS, STATA) for statistical analyses.

## Stepwise procedure

**Figure 2:**
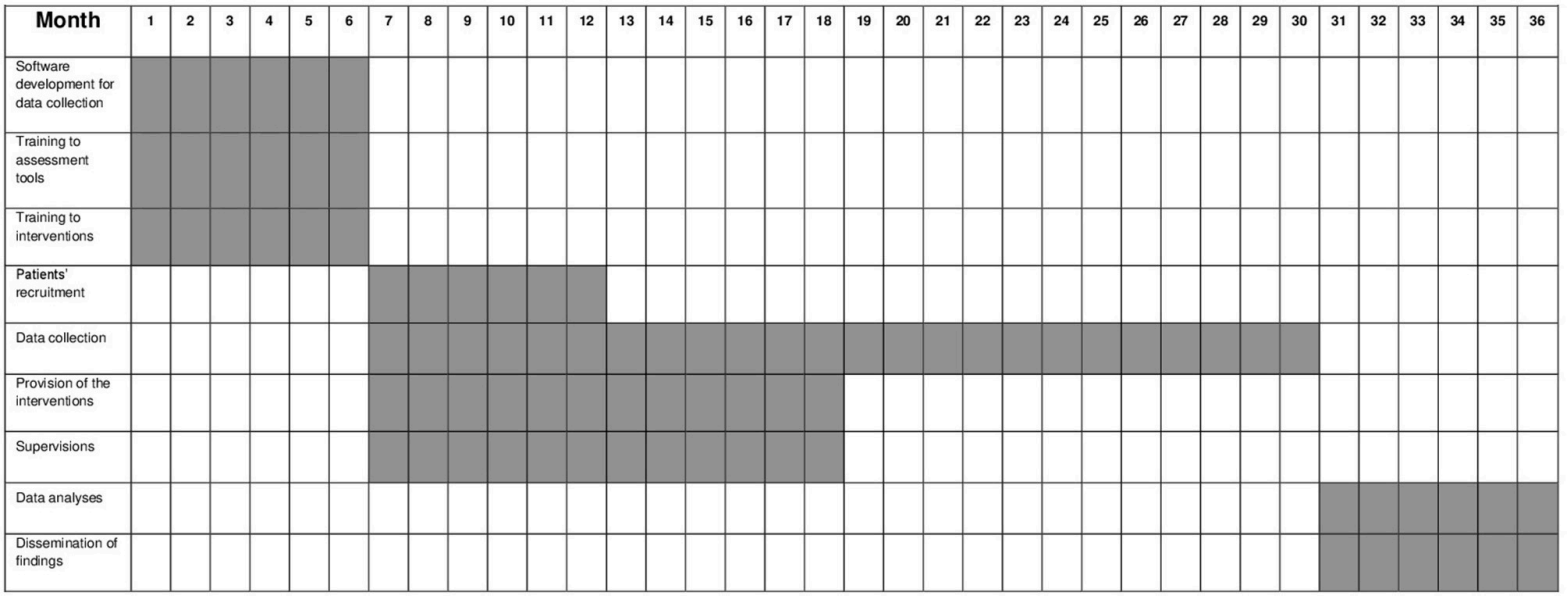
Stepwise procedure.

From month 1 to 6, the following phases have been carried out: (1) development of the work plan for data collection in each participating center; (2) training course for mental health professionals on the interventions; (3) training course for researchers on assessment tools. From month 7 to 12, patients' recruitment has been implemented. From month 7 to 18, interventions are being provided to participating patients. From month 7 to 30, patients' follow-up assessments are being made. From month 31 to 36, statistical analyses will be performed as well as findings will be disseminated through papers and participation in conferences. The stepwise procedure is summarized in Figure 2.

## Discussion

Unhealthy and unbalanced diet, physical inactivity, smoking, and excessive alcohol or substance use are very common in people with severe mental disorders (12, 56–60). All these factors contribute to the excess mortality found in those people compared to the general population. It is an ethical and a clinical priority to develop and implement effective strategies for modifying and eliminating those risk factors (61, 62).

One possible strategy is the provision of psychosocial integrated interventions specifically tailored on the physical needs of patients with severe mental disorders (63). In fact, as recently pointed out by De Rosa et al. (25), although several psychosocial interventions have been developed on different aspects of unhealthy lifestyle behaviors, their feasibility has not been evaluated in routine care.

The research protocol presented herein proposes an innovative approach to manage unhealthy lifestyle behaviors in patients with severe mental disorders and to promote a pattern of change. In particular, the main novelties of the LIFESTYLE psychosocial group intervention are: (1) the integration of classic psychoeducational with motivational and cognitive-behavioral therapy techniques; (2) the adoption of a comprehensive approach, addressing not only unhealthy diet and overweight, but focusing on all aspects of unhealthy lifestyles; (3) the provision of the intervention to a mixed diagnostic group of patients; (4) the inclusion of the recommendations and/or guidelines on the management of physical health in people with mental disorders produced by the World Health Organization (33, 34), the European Association for Study of Diabetes (35), the European Society of Cardiology (36) and the European Psychiatric Association (37).

The inclusion of the motivational interview is one of the major strengths of the LIFESTYLE intervention. Several studies (64–66) have proven that increasing motivation is a successful strategy in the whole medicine for supporting medication adherence (67) or weight loss (68). During the first session of the LIFESTYLE psychosocial group intervention, each participant is required to identify a personal lifestyle goal that should be realistic and achievable in a relatively short period of time. Patients are empowered to determine their lifestyle goals and the intervention can contribute to their specific recovery goals.

Moreover, it is well known that patients with severe mental disorders are at high risk of developing physical diseases and receiving late diagnoses due to the underestimation of somatic comorbidity. Through the psychoeducational approach, we will provide patients with information on main physical complaints and on how to detect them. In particular, through focus groups with the participation of expert clinicians and/or users and carers, we have developed written materials (such as leaflets and brief manuals) targeted to the physical needs of people with severe mental disorders that will be distributed to participants whenever necessary (69–71).

The LIFESTYLE trial adopted a comprehensive approach focusing on all aspects of unhealthy behaviors, since the increased morbidity and mortality of patients with severe mental disorders is due to the interaction of several risk factors. In particular, dietary pattern and sedentary behaviors are easier to change and therefore will be addressed in the first two sessions of the LIFESTYLE intervention, when patients' drop-out risk is higher. Another important topic is the reduction of smoking, which is frequent in patients with severe mental disorders (72–74). The reduction in smoking habit is not the primary outcome of the study, and for this reason we did not propose to patients specific pharmacological (e.g., nicotine patches, nicotine gum, bupropion, or nortriptyline) or non-pharmacological strategies (e.g., cognitive-behavioral therapy group targeted on substance abuse) for supporting smoking cessations, as already done in previous studies (26). Nevertheless, we expect a reduction in the number of cigarettes smoked per day, as a consequence of the global lifestyle improvement. Also, patients with severe mental disorders frequently have risky sexual behaviors, which are often due to the presence of cognitive deficits and manic symptoms, and represent the cause of many sexual transmitted diseases. Moreover, the sexual health of patients with severe mental disorders is often neglected by clinicians who do not routinely ask to patients about their sexual life (75, 76). The circadian rhythms are frequently altered due to polarity switch of mood in affective disorders or to the non-adherence to psychotropic medications. Therefore, in our intervention a specific session dealing with those aspects has been included, since these aspects must be taken into account for achieving a global improvement in lifestyle.

Non-adherence to medications is responsible for a variety of clinical and social problems, such as relapses, hospitalization, reduction in quality of life and in personal functioning [(77, 78)]; thus, improving medication compliance represents another important challenge addressed during the intervention. Since the LIFESTYLE intervention is patient-centered and motivational-led (78), it has been developed to fulfil the unmet needs of patients with severe mental illness, including the improvement of treatment adherence.

As regard our primary outcome measure, the BMI has been selected since it is a reliable and feasible anthropometric parameter to be analyzed. Of course, there are many factors which can have an impact on BMI changes, such as the pharmacological treatment (e.g., in the case of patients receive second generation antipsychotics) or other physical comorbidities (such as, endocrine diseases). We will be able to control for these possible confounders through multivariable statistical approach. Moreover, patients are required to identify a personal lifestyle goal to be achieved, and—by definition—such goal can be different for each participant, e.g., days spent in physical activities; drinking at least 2 l of water per day; reducing the number of cigarettes smoked per day; etc. and not easily measurable. In the experimental intervention, it has been included a brief session of moderate physical active at the end of each meeting, therefore we test the hypothesis that BMI can be reduced, even if the personal goal selected by the patient is related to smoking pattern or to other aspects of the lifestyle. As observed by Speyer et al. (22, 23), adopting an heterogeneous intervention, when every patient can choose the personal goal to be reached, can be the right choice for changing a complex phenomenon as lifestyle. The prevalence of unhealthy lifestyle behaviors is a transdiagnostic phenomenon and we decided not to tailor the intervention to a specific group of patients in order to involve the highest number of patients as possible. As recently pointed out (25) when the patient population is selected on the basis of the diagnoses, there are no data proving the higher efficacy of the lifestyle intervention.

In this study, we aim to evaluate the cost-effectiveness of the interventions, since only Verhaeghe et al. (79) found that the intervention is cost-effective only in men with a significant BMI reduction. For improving the dissemination of psychosocial interventions targeted on healthy lifestyle behaviors, it is necessary to prove that these interventions are cost-saving. In order to improve feasibility of the interventions in the clinical routine care, we decided not to include dieticians or physical trainers, since the involvement of health professionals different from psychiatrists or psychologists could hamper the dissemination of the interventions in the routine practice. Of course, this hypothesis should be further tested in multicentric studies involving different European countries, since it could be that workload and multidisciplinary staffs include different health professional according to the organization of the National health care. At least in Italy, the inclusion of specific health professionals such as dieticians and physical trainers is not the routine for the mental health care. A critical element in our research protocol is the attrition rate from the two intervention groups. As already pointed out in other studies on lifestyle psychosocial interventions, such as the CHANGE (22, 23) and CAPICOR (21) trials, the attrition rate ranges from 5 to 40% and we expect an attrition rate of at least 15%. Nevertheless, in order to guarantee attendance, patients will continue to be in contact with their referring clinicians during the provision of the intervention, and will receive phone or e-mail reminders for each session of the intervention. Moreover, the active involvement of patients as “experts” could further motivate them to attend the sessions during the intervention. This project represents the first multicentric randomized head-to-head comparison trial for evaluating the efficacy of psychosocial interventions targeting unhealthy lifestyle in people with severe mental disorders in Italy. In particular, we aim to test the efficacy of two different interventions targeting lifestyle behaviors. In particular, considering that one of the main critical issue is related to the optimal duration of the interventions, this aspect will be evaluated in our trial since we will test the impact of the “duration of the intervention”, as one of the possible mediator or moderator of the efficacy of the interventions.

The trial has some limitations, which must be acknowledged. A first limitation is the selection bias of patients accepting to participate to the LIFESTYLE trial. In fact, it could be that only highly motivated patients accept to take part to the study and will continue to attend all scheduled sessions. Another possible limitation is the use of manuals for clinicians carrying out the intervention. While this methodological choice was made in order to increase external validity and reliability of the interventions, it may have limited the generalizability of the intervention to other patients. Moreover, the two interventions are different in terms of frequency of contact with the mental health professionals. This aspect will be considered as a potential confounder for the effectiveness of the intervention. Finally, another possible limitation of the study is the global duration of the intervention. In fact, Naslund et al. (80) recently highlighted that lifestyle interventions of ≥12-months duration appear to be more effective compared to those lasting ≤6-months. Moreover, other studies have found a modest or small effect for short-term interventions, suggesting that longer interventions should be studied. On the other hand, long approaches are associated with a higher drop-out rate of patients who may have difficulties in attending structured long-term approaches (81–84). Therefore, we will explore the efficacy and the effectiveness of a medium-term intervention with multiple follow-up assessments.

## Conclusions

In people with severe mental disorders, unhealthy lifestyle is very frequent and is associated with poor outcomes. Therefore, there is the need to develop and test the efficacy of new interventions for changing unhealthy behaviors. We hope that the study protocol presented herein will help to fill this gap and improve physical health of patients with severe mental disorders.

## Added value of this study

To our knowledge, this is the first multicentric randomized head-to-head comparison trial of behavioral change intervention targeting unhealthy lifestyle in people with severe mental disorders in Italy.

The main novelty of our approach is the integration of classic psychoeducation with motivational and cognitive-behavioral therapy techniques. Both interventions have been developed following the recommendations and guidelines on the management of physical health in people with mental disorders produced by the World Health Organization (33, 34), the European Association for Study of Diabetes (35), the European Society of Cardiology (36), and the European Psychiatric Association (37). Moreover, our interventions did not target only one aspect of unhealthy lifestyle behaviors, but aim to promote a global change in lifestyle behaviors, which should become more oriented to the healthy living approach. We believe that pursuing such aim should be more useful, feasible and acceptable in patients with severe mental disorders, rather than the achievement of changing just one among all unhealthy behaviors. Creating and disseminating the culture of “living healthy” should be useful to guarantee a long-term change in patients' behaviors and for reducing significantly the mortality gap with the general population.

## Ethics statement

This study was carried out in accordance with the recommendations of Ethical Committee of the University of Campania Luigi Vanvitelli. The protocol was approved by the Ethical Committee of the University of Campania Luigi Vanvitelli. All subjects gave written informed consent in accordance with the Declaration of Helsinki.

## Author contributions

GS, AF, ML, and MM designed the study and wrote the protocol. AF, VDV, ML, CDR, CM, GB, VG, MS, and GS organized the training and supervisions for mental health professionals, and coordinated the activities with participating mental health centers. GS, ML, LS, and BP managed the literature searches and analyses. MB, MA, FP, LDO, and GDL coordinated the study in their center. The working group includes: GB, CDR, VG, CM, and MS (Department of Psychiatry, University of Campania Luigi Vanvitelli, Naples); AF, LL, and FV (University of Bari Aldo Moro); MBM, PC, and DZ (University of Genova); FL, SP, and DS (University of L'Aquila); CC, ED, and MTA (University of Pisa); EB, CN and AS (University of Rome Tor Vergata). All working group participants contributed to the development of the training materials and participated to the training course for the interventions and for the assessment tools.

### Conflict of interest statement

The authors declare that the research was conducted in the absence of any commercial or financial relationships that could be construed as a potential conflict of interest. The reviewer IE and handling Editor declared their shared affiliation.
